# Case Report: Bladder perforation in an extremely preterm neonate secondary to multifactorial etiology

**DOI:** 10.3389/fped.2026.1912735

**Published:** 2026-10-02

**Authors:** Ismail Alwadani, Tahseen Elwahsh, Batool Albahrani

**Affiliations:** 1 Neonatal Intensive Care Department, Johns Hopkins Aramco Healthcare, Dhahran, Saudi Arabia; 2 Pediatric Department, Johns Hopkins Aramco Healthcare, Dhahran, Saudi Arabia

**Keywords:** acute kidney injury, ascites, bladder perforation, neonate, umbilical catheter, urinary catheter

## Abstract

**Background:**

Neonatal bladder perforation is a rare but life-threatening condition associated with significant morbidity if not promptly recognized.

**Case presentation:**

We report the case of an extremely preterm male infant born at 26 weeks of gestation who developed bladder perforation on day 15 of life, presenting with abdominal distension, decreased urine output, and respiratory distress. The diagnosis was confirmed by fluoroscopic voiding cystourethrogram demonstrating contrast extravasation into the peritoneal cavity. The etiology was multifactorial, involving extreme prematurity, umbilical arterial catheterization, repeated urinary catheter insertions, and heavy sedation. Initial conservative management with urinary catheterization and broad-spectrum antibiotics was followed by successful surgical repair via exploratory laparotomy on day 23 of life.

**Conclusion:**

This case highlights the importance of maintaining a high index of suspicion for bladder perforation in extremely preterm neonates presenting with ascites and sepsis-like features, particularly in the presence of multiple iatrogenic risk factors. Early diagnosis and timely intervention are critical for optimizing outcomes.

## Introduction

Urine extravasation into the peritoneal cavity resulting from bladder perforation is a rare complication in neonates, carrying a substantial mortality risk if not recognized early (1, 2). Both congenital and acquired factors contribute to its etiology (3). The clinical presentation is frequently nonspecific — abdominal distension, respiratory deterioration, and oliguria — which can delay diagnosis. The most reliable confirmatory investigation is a voiding cystourethrogram (VCUG) (4). What makes the present case unique is the constellation of multiple concurrent iatrogenic and physiological risk factors in a 26-week-gestation infant (extremely preterm, defined as birth before 28 weeks of gestation): umbilical arterial catheterization, repeated urinary catheterizations, extreme prematurity, and prolonged heavy sedation — none of which alone is sufficient to explain the perforation, but together represent a compounding multifactorial mechanism not previously described in this combination. We discuss the clinical course, diagnostic workup, and management, and draw lessons for neonatal intensive care practice.

## Case description

A male infant was born at 26 weeks of gestation via emergency cesarean section to a 26-year-old gravida 2 para 1 mother, indicated for chorioamnionitis. Antenatal ultrasound had demonstrated oligohydramnios and a retroplacental clot. Birth weight was 890 g. The infant was flat, apneic, and cyanotic at delivery, with Apgar scores of 4 and 8 at 1 and 5 min, respectively. Resuscitation was performed per Neonatal Resuscitation Program (NRP) protocol, requiring intubation in the delivery room. The infant was admitted to the neonatal intensive care unit (NICU) with diagnoses of extreme prematurity and respiratory distress syndrome (RDS). He received two doses of endotracheal surfactant and was commenced on high-frequency oscillatory ventilation (HFOV), intravenous antibiotics, and total parenteral nutrition. Umbilical arterial and venous catheters were successfully inserted on day 1, each in a single attempt, with radiographic confirmation of satisfactory catheter tip position; the UVC remained in place for 10 days. No relevant family or psychosocial history was reported.

On day 2, the infant developed hypotension requiring inotropic support with intravenous dopamine and epinephrine, and was sedated with intravenous midazolam and fentanyl infusions. On day 3, acute kidney injury (AKI) supervened, with azotemia, hyponatremia, and hyperkalemia. An accidental midazolam overdose was identified, having occurred over the preceding 32 h, with the administered dose approximately double the usual maximum recommended dose; hyperkalemia was managed per protocol and a non-balloon, 3.5 Fr urinary catheter was inserted to a length of 4 cm, following two insertion attempts, with no resistance or bleeding noted, though no urine output was initially recorded. By day 4, a polyuric phase of AKI was evident, and renal ultrasound showed bilateral mild-to-moderate hydronephrosis. Renal function and urine output improved by day 6, with trophic feeds initiated. On day 8, the infant was extubated to non-invasive ventilation; the umbilical arterial catheter and urinary catheter were removed as an elective step in de-escalating invasive monitoring coinciding with overall clinical improvement, and feeds were advanced.

On day 15, the infant developed progressive abdominal distension and respiratory distress with decreased urine output, in the absence of gastrointestinal manifestations. Abdominal examination revealed marked distension with associated respiratory distress; vital signs at presentation showed desaturation, tachycardia, and hypotension. Abdominal plain radiograph showed a paucity of bowel gas with diffuse opacification consistent with ascites (Figure 1). Pediatric surgery was consulted for suspected abdominal compartment syndrome. Paracentesis was performed and a Penrose drain was placed in the right lower quadrant, yielding approximately 200 mL of clear, pale-yellow fluid (Figure 2). Chemical analysis of the peritoneal fluid revealed urea, creatinine, and electrolyte values comparable to those in serum, consistent with a urinary origin (Table 1). A subsequent fluoroscopic voiding cystourethrogram (VCUG) confirmed contrast extravasation from the bladder into the peritoneal cavity, establishing the diagnosis of bladder perforation (Figure 3). Concurrent bladder ultrasound demonstrated an underdistended bladder with no renal anatomical malformation. External genital examination revealed normal male genitalia with no congenital anomalies. Spontaneous voiding pattern and post-void residual assessment were not specifically documented in the medical record and are addressed further under Differential Diagnosis below. A second urinary catheter, together with a peripherally inserted central catheter, was placed at this time for ongoing bladder drainage and vascular access; the bladder had therefore been catheterized on two separate occasions during the clinical course (day 3–8, and again from day 15), consistent with the repeated catheterization cited among the contributing factors below. The etiology was attributed to a combination of risk factors: extreme prematurity, umbilical arterial catheterization, multiple urinary catheter insertions, and prolonged heavy sedation.

**Figure 1:**
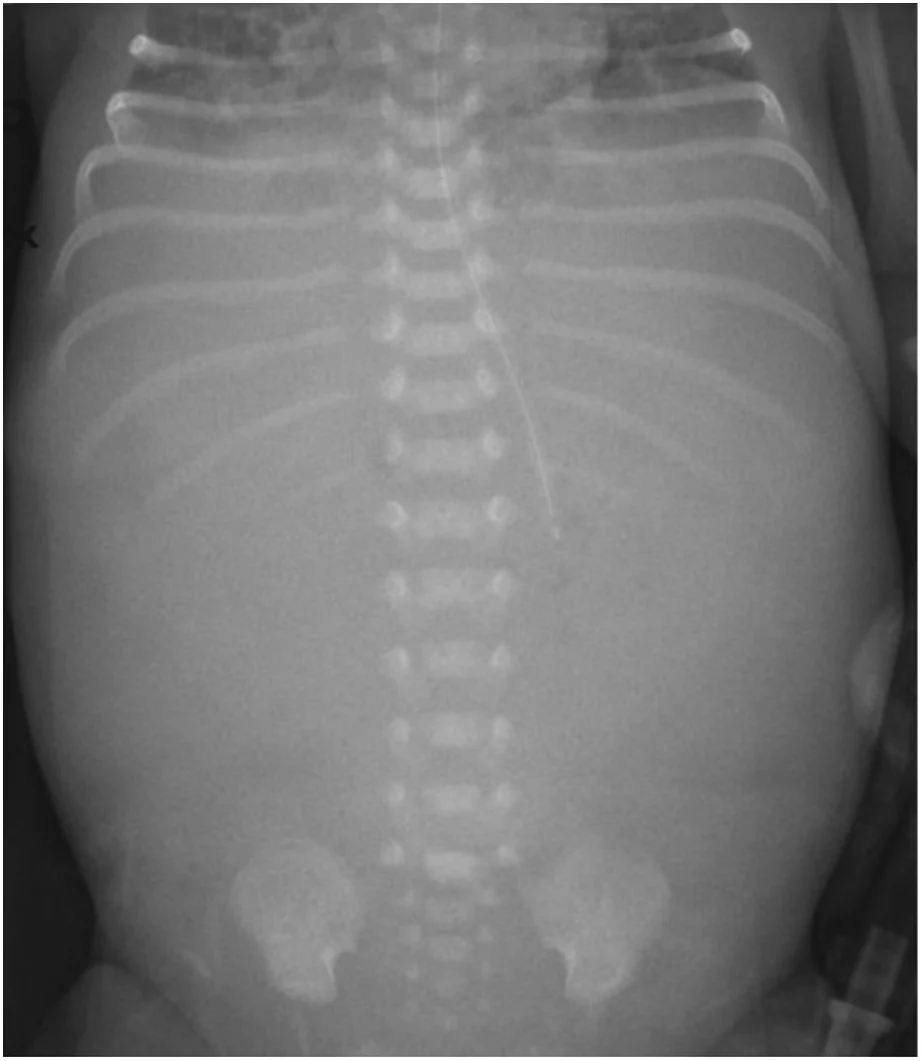
Abdominal plain radiograph showing paucity of bowel gas with diffuse opacification consistent with ascites.

**Figure 2:**
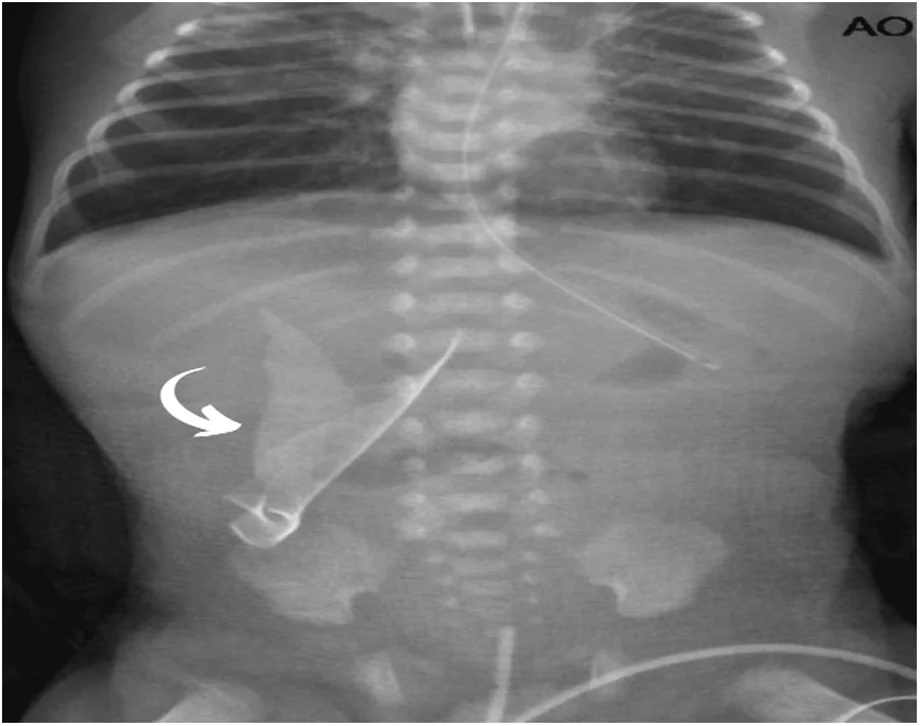
Post-penrose drain insertion abdominal radiograph. The urinary bladder catheter and the Penrose drain (arrow), positioned in the right lower quadrant to decompress the peritoneal cavity, are both *in situ*. Image quality is limited by diffuse abdominal opacity related to residual ascites; despite this, there is interval improvement in lung aeration and slight improvement in abdominal distension following drain insertion.

**Figure 3:**
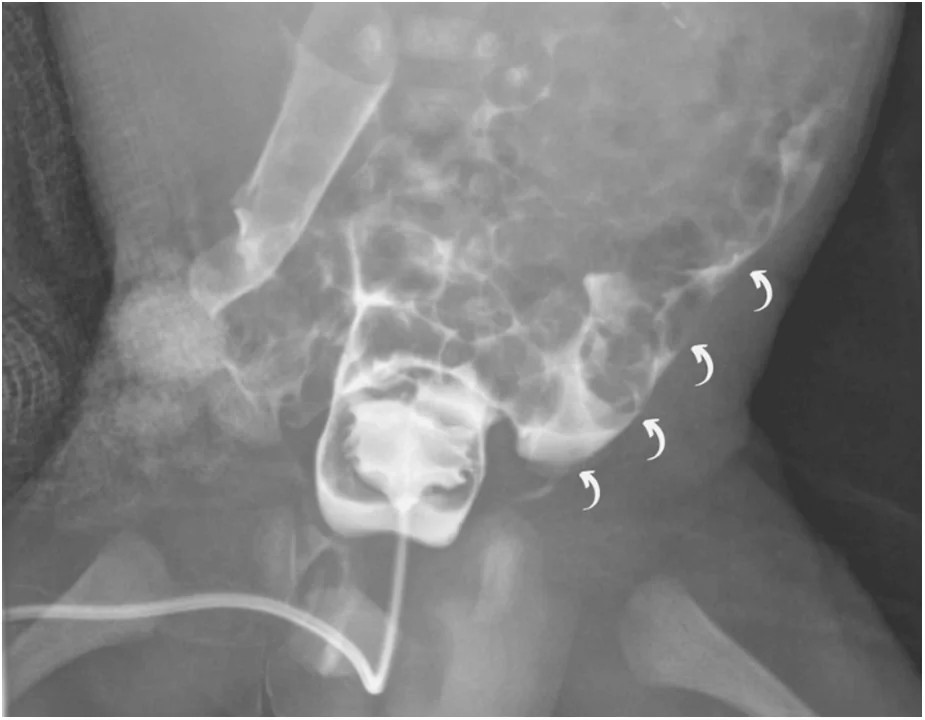
Fluoroscopic voiding cystourethrogram showing contrast extravasation into the peritoneal cavity, confirming bladder perforation (arrow).

**Table 1:** Peritoneal fluid versus serum analysis.

Parameter	Peritoneal Fluid	Serum Value	Reference Range
BUN (mg/dL)	64	45	4–12
Creatinine (mg/dL)	1.8	1.7	0.66–1.25
Sodium (mEq/L)	98	125	135–145
Potassium (mEq/L)	12.9	8.2	4.1–5.3
Albumin (g/dL)	<1.0	3.5	2.8–4.4
Glucose (mg/dL)	73	104	73–178
White blood cells (K/cumm)	20	20.9	6.0–17.5
Red blood cells (M/cumm)	740	3.63	3.1–4.5

Comparison of peritoneal fluid and serum laboratory values. BUN, blood urea nitrogen. Near-identical BUN and creatinine concentrations in peritoneal fluid and serum confirm the urinary origin of the ascites.

## Diagnostic assessment, therapeutic intervention, follow-up, and outcomes

The diagnosis of bladder perforation was established by a combination of clinical, biochemical, and radiological findings. The presenting triad of abdominal distension, respiratory deterioration, and oliguria in the context of multiple iatrogenic risk factors prompted urgent evaluation. Peritoneal fluid analysis (Table 1) demonstrated near-identical urea, creatinine, and electrolyte concentrations to those in serum, establishing a urinary origin. No formal urinalysis or urine microscopy was performed or documented following the diagnosis of bladder perforation; the peritoneal fluid biochemistry described above supports a urinary origin of the ascites but is not equivalent to urinalysis and does not substitute for it in assessing for hematuria or leukocyturia. Fluoroscopic VCUG confirmed contrast extravasation into the peritoneal cavity (Figure 3), consistent with bladder perforation. Bladder ultrasound revealed an underdistended bladder with no underlying anatomical malformation, excluding a congenital cause.

## Differential diagnosis

Given the patient's male sex, history of oligohydramnios, and presentation with urinary ascites, posterior urethral valves (PUV) and other causes of bladder outlet obstruction (BOO) were considered as an alternative or contributing explanation, since chronic outlet obstruction with elevated intravesical pressure is a recognized mechanism of spontaneous bladder or renal fornix rupture in this population (5). Several features argued against high-grade PUV/BOO as the primary mechanism in this case: bladder ultrasound at the time of perforation demonstrated an underdistended, rather than distended or trabeculated, bladder, with no upper-tract dilatation or renal anatomical malformation; and the perforation occurred in the temporal context of recent urinary catheterization and heavy sedation, both independently recognized causes of bladder wall injury. Other differentials considered included primary sepsis/necrotizing enterocolitis (rendered unlikely by the absence of gastrointestinal signs and by peritoneal fluid biochemistry closely matching serum urea, creatinine, and electrolytes rather than a septic or enteric profile), chylous ascites (excluded by the biochemical profile and fluid appearance), and bowel perforation (excluded by the absence of free intraperitoneal air on imaging and by the urinary rather than enteric biochemical signature of the fluid). External genital examination revealed normal male genitalia with no congenital anomalies, arguing further against an obstructive congenital anomaly such as PUV. However, a dedicated voiding-phase assessment of the posterior urethra and post-void residual measurement were not done, and intraoperative cystoscopy was not performed. We therefore acknowledge that PUV/BOO cannot be definitively and formally excluded on the basis of the data available; this is noted as a limitation, with follow-up voiding assessment recommended if not already performed.

## Therapeutic intervention

Given the infant's medical instability — requirement for HFOV and body weight of 870 g at presentation — initial management was conservative, comprising continuous urinary catheter drainage and broad-spectrum intravenous antibiotics (cefepime and vancomycin, per unit policy, with dosing adjusted for renal function). Antibiotics were subsequently adjusted based on culture and sensitivity results. The Penrose drain was maintained to decompress the peritoneal cavity and facilitate respiratory improvement.

By day 23 of life, the infant had stabilized on conventional ventilation with a weight of 830 g, at which point exploratory laparotomy was performed. Intraoperatively, three small perforations were identified at the posterior right dome of the bladder. The urachus was closed with no evidence of patency, the intra-abdominal umbilical vessel remnants were unremarkable with no inflammation or adherence to the bladder dome, and the bladder mucosa showed no evidence of trabeculation or other signs of chronic obstruction. Neither intraoperative cystoscopy nor a temporizing vesicostomy was performed. These perforations were repaired with absorbable sutures and the peritoneal drain was removed. Postoperatively, the infant was monitored closely for peritonitis and sepsis.

## Follow-up and outcomes

The postoperative course was uneventful. Abdominal distension resolved progressively and renal function normalized. The urinary catheter was removed on postoperative day 10. Follow-up imaging confirmed no evidence of residual urinary leakage. The infant was discharged home in good condition and was followed up jointly by pediatric nephrology and urology until one year of age. Post-discharge renal/bladder ultrasound was performed on two occasions and showed normal findings on both, serum creatinine and blood pressure remained within normal limits throughout follow-up, and there was no documented history of urinary tract infection. On outpatient follow-up, the infant demonstrated normal growth and neurodevelopmental progress. Table 2 summarize timeline of key clinical events by day of life.

**Table 2:** Timeline of clinical events.

Day of Life	Key Clinical Events
DOL 1	Born at 26 weeks GA via emergency cesarean section; flat and apneic at birth; intubated in delivery room; NICU admission for extreme prematurity and RDS; two doses of intratracheal surfactant; HFOV commenced; IV antibiotics started; umbilical arterial and venous catheters inserted; TPN initiated.
DOL 2	Hypotension developed; IV dopamine and epinephrine infusions started; IV midazolam and fentanyl infusions commenced for sedation.
DOL 3	Acute kidney injury onset; azotemia, hyponatremia, and hyperkalemia noted; accidental midazolam overdose identified (32-hour exposure); hyperkalemia managed per protocol; urinary catheter inserted — no urine output recorded.
DOL 4	Polyuric phase of AKI; renal ultrasound: bilateral mild-to-moderate hydronephrosis.
DOL 6	Renal function and urine output improved; trophic enteral feeds initiated.
DOL 8	Extubated to non-invasive ventilation; umbilical arterial catheter and urinary catheter removed; feeding advanced.
DOL 15	New-onset abdominal distension, decreased urine output, and respiratory distress; abdominal x-ray: ascites; paracentesis performed; Penrose drain inserted — 200 mL yellow fluid drained; peritoneal fluid analysis consistent with urinary origin; urinary catheter and PICC line inserted.
DOL 16	Fluoroscopic VCUG confirmed bladder perforation with contrast extravasation; bladder ultrasound: underdistended bladder, no renal malformation. Conservative management commenced: urinary catheterization+broad-spectrum antibiotics.
DOL 17–22	Continued conservative management; clinical monitoring for peritonitis and sepsis; antibiotics adjusted per culture results.
DOL 23	Weight 830 g; stable on conventional ventilation. Exploratory laparotomy performed; three small perforations identified at the posterior right dome of the bladder; repaired with absorbable sutures; peritoneal drain removed.
POD 10 (DOL 33)	Urinary catheter removed; follow-up imaging confirmed no urinary leakage; abdominal distension resolved; renal function normalized.
Discharge	Infant discharged home in good condition; outpatient follow-up confirmed normal growth and development.

Timeline of key clinical events by day of life. DOL, day of life; GA, gestational age; RDS, respiratory distress syndrome; HFOV, high-frequency oscillatory ventilation; TPN, total parenteral nutrition; AKI, acute kidney injury; VCUG, voiding cystourethrogram; PICC, peripherally inserted central catheter; POD, postoperative day.

## Discussion

Neonatal bladder perforation is uncommon. Known risk factors include posterior urethral valves, neurogenic bladder, congenital bladder diverticulum, detrusor areflexia, umbilical arterial catheterization, and urethral catheterization (1, 5). Spontaneous bladder rupture has also been documented in association with opioid-induced urinary retention and severe hypoxia (6). In the present case, no single factor was solely responsible; rather, a convergence of extreme prematurity, umbilical arterial catheterization, multiple Foley catheter insertions, and prolonged high-dose sedation — including an accidental midazolam overdose of approximately twice the usual maximum recommended dose sustained over 32 h — created cumulative mechanical and pharmacological stress on an immature bladder wall.

Beyond these four proposed factors, perinatal hypoxia-ischemia merits consideration as an additional contributor. This infant experienced chorioamnionitis, emergency cesarean delivery, low Apgar scores, and a requirement for high-frequency oscillatory ventilation, together with acute kidney injury in the first days of life — a constellation consistent with perinatal circulatory stress. Hypoxia-ischemia can redirect blood flow away from non-vital viscera, including the bladder, and can impair detrusor and sacral micturition center function, potentially rendering the bladder wall more vulnerable to mechanical and pharmacological insults and contributing to oliguria-related overdistension. Chorioamnionitis-associated inflammation may similarly weaken bladder wall integrity, and comorbidities of extreme prematurity such as hypotension and acute kidney injury may have further compromised tissue perfusion and healing capacity. Table 3 summarizes the proposed contributing factors and the evidence for and against each, including posterior urethral valves/bladder outlet obstruction, which was considered and is discussed above under Differential Diagnosis.

**Table 3:** Proposed contributing factors to bladder perforation in this case, weighed against available evidence.

Proposed Factor	Supporting Evidence in This Case	Alternative/Confounding Considerations
Extreme prematurity (26 weeks, 890 g)	Detrusor immaturity and thin bladder wall are recognized vulnerabilities of the extremely preterm bladder	Present in all extremely preterm infants; most do not develop perforation, so prematurity alone is insufficient
Umbilical arterial catheterization	UAC is a recognized cause of vesical ischemic injury in neonates (1, 4)	Insertion successful on first attempt for each catheter, with radiographically confirmed good tip position; UVC in place for 10 days
Repeated urinary catheterization	Catheter placed on two separate occasions (day 3–8 and from day 15); Foley catheterization is a described cause of bladder perforation (2, 3)	First catheterization (day 3): non-balloon, 3.5 Fr catheter, inserted to 4 cm over two attempts, with no resistance or bleeding noted. Second catheterization (day 15) details not fully documented
Prolonged heavy sedation (midazolam overdose)	Sedation/opioid-induced urinary retention with bladder overdistension is a described mechanism (6)	Administered dose was approximately double the usual maximum recommended dose, sustained over 32 h, supporting a substantive (not minor) contribution
Perinatal hypoxia-ischemia	Chorioamnionitis, low Apgar scores, HFOV requirement, and AKI suggest perinatal circulatory stress	Not directly measured (e.g., cord gas/lactate not reported)
Posterior urethral valves/bladder outlet obstruction	Underdistended bladder and normal renal anatomy on ultrasound argue against high-grade obstruction	External genitalia exam normal, with no congenital anomalies. Voiding-phase urethral imaging and post-void residual not separately documented; cannot be fully excluded

Neonatal urinary ascites constitutes a life-threatening condition because the peritoneal membrane acts as an autodialyzer, reabsorbing urea and creatinine and facilitating electrolyte exchange. The resulting biochemical pattern — elevated serum urea and creatinine, hyponatremia, and hyperkalemia — should prompt strong suspicion of urinary ascites in any neonate presenting with abdominal distension (7–11). When accompanied by a sepsis-like picture, the rarity of this diagnosis can lead to delayed recognition (12).

Imaging plays a central diagnostic role. Ultrasound can detect ascites and upper tract dilatation; VCUG remains the gold standard for localizing the site of leakage and characterizing any underlying urinary tract abnormality (13). In the present patient, VCUG unequivocally demonstrated extravasation from the posterior right dome of the bladder, guiding both the surgical approach and the intraoperative findings.

Regarding management, several authors advocate aggressive open repair (12); however, conservative treatment with catheter drainage and antibiotics has been successfully employed in hemodynamically unstable patients (14). In this case, a staged approach was adopted: initial stabilization conservatively, followed by definitive surgical repair once the infant reached an appropriate weight and respiratory stability. This strategy proved safe and effective.

Long-term consequences of bladder perforation can include impaired bladder emptying, bladder scarring, bilateral hydronephrosis, renal impairment, and chronic kidney disease (15). The absence of these sequelae at follow-up in this patient is reassuring and likely reflects the timely diagnosis and complete surgical repair achieved.

Compared with previously reported cases, the present infant represents one of the most extreme examples of prematurity and low birth weight associated with neonatal bladder perforation reported to date. Birs et al. (15), for example, described iatrogenic bladder perforation diagnosed with delay in a term infant (37 weeks’ gestation, birth weight 3,528 g) — a fundamentally different physiological context from the present 26-week, 890-g infant, underscoring that the mechanism and risk profile of iatrogenic bladder injury differ substantially across gestational ages. Vasdev et al. (14) reported four neonates with urinary ascites secondary to bladder rupture, noting normal antenatal genitourinary imaging in all cases and management ranging from conservative treatment (*n* = 3) to surgical repair (*n* = 1); the present case extends this staged conservative-to-surgical paradigm into the extremely preterm population, where hemodynamic and respiratory instability further complicate the timing of surgical intervention.

Strengths of this report include the detailed documentation of a genuinely rare entity in one of the highest-risk neonatal populations, with comprehensive imaging evidence and surgical confirmation. Limitations include: the inability to fully and formally exclude posterior urethral valves/bladder outlet obstruction on the basis of the available imaging and examination record; incomplete documentation of second-catheterization (day 15) details and insertion-related complications for the urinary catheter (the first urinary catheterization is documented as a non-balloon, 3.5 Fr catheter, inserted to 4 cm over two attempts; UAC/UVC placement was confirmed on the first attempt with satisfactory tip position and a 10-day UVC duration); the absence of a formal urinalysis or urine microscopy at the time of diagnosis, such that hematuria or leukocyturia could not be formally assessed beyond the peritoneal fluid biochemistry; and the absence of formal urodynamic or voiding-function assessment during the one-year follow-up period, despite reassuring serial renal/bladder ultrasound, creatinine, and blood pressure results. These gaps reflect the constraints of retrospective case documentation and are acknowledged transparently. The key take-away lesson is that in extremely preterm neonates with multiple iatrogenic interventions who develop abdominal distension and oliguria, bladder perforation should be included in the differential diagnosis and investigated promptly with peritoneal fluid biochemistry and VCUG.

## Patient perspective

The infant's parents were kept fully informed throughout the admission. They described the experience as extremely distressing, particularly during the acute deterioration on day 15 when the diagnosis of bladder perforation was established. The family expressed that clear, regular communication from the neonatal and surgical teams — explaining each diagnostic step and the rationale for the staged management approach — was essential in helping them cope with the uncertainty. The parents consented to surgical repair and reported feeling well-supported during the postoperative period. At follow-up, they noted their child's normal developmental progress with relief and gratitude, and agreed to share their son's case to help advance clinical awareness of this rare but serious condition.

## Data Availability

The original contributions presented in the study are included in the article/supplementary material, further inquiries can be directed to the corresponding author.
